# Investigating the safety of physical rehabilitation with critically ill patients receiving vasoactive drugs: An exploratory observational feasibility study

**DOI:** 10.1371/journal.pone.0318150

**Published:** 2025-02-13

**Authors:** Huw R. Woodbridge, Caroline M. Alexander, Stephen J. Brett, David B. Antcliffe, Ee Lyn Chan, Anthony C. Gordon

**Affiliations:** 1 Imperial College Healthcare National Health Service Trust, London, United Kingdom; 2 Division of Anaesthetics, Pain Medicine and Intensive Care, Imperial College London, London, United Kingdom; 3 Department of Surgery and Cancer, Imperial College London, London, United Kingdom; 4 Maidstone and Tunbridge Wells National Health Service Trust, Kent, United Kingdom; Albert Einstein College of Medicine, UNITED STATES OF AMERICA

## Abstract

**Background:**

Physical rehabilitation of critically ill patients may improve physical outcomes; however, the relative benefits and risks with patients requiring vasoactive drugs is currently unknown. A feasibility study is needed to inform the design of a future trial required to address this issue.

**Methods:**

A two-phase exploratory observational feasibility study was carried out:

1. A retrospective study to clarify the current practice of rehabilitation with patients receiving vasoactive drugs to inform future trial interventions and design.
2. A prospective study exploring recruitment and outcome measurement. Intensive care patients receiving vasoactive drugs were recruited and asked about the acceptability of a future trial. The feasibility of using an adverse event tool was measured during rehabilitation. Patients were followed up after 60 days to describe the feasibility of measuring outcomes for a future trial.

**Results:**

Retrospective study (n = 78): Twenty-one percent of patients took part in physical rehabilitation whilst receiving vasoactive drugs. Of 321 days with vasoactive drugs administered, physical rehabilitation occurred on 27 days (8%).

Prospective study (n = 40): Eighty-one percent of participants indicated acceptability of being recruited into a future trial (n = 37). Eighty-eight percent of clinicians found it acceptable to randomise patients into either early rehabilitation or standard care. The adverse event tool was implemented by researchers with 2% loss of information. Finally, a 100% follow-up rate at day 60 was achieved for mortality outcomes. Follow-up rates were 70% for the EQ-5D (5 level), 65% for the World Health Organisation’s Disability Assessment Schedule 2.0 and RAND 36-item Health Survey 1.0 and 26% for the 6-minute walk test.

**Conclusions:**

This study found a low frequency of physical rehabilitation occurring with intensive care patients receiving vasoactive drugs. A high proportion of clinicians and patients found a future RCT within this patient group acceptable. Mortality and patient-reported outcomes were the most feasible to measure.

## Introduction

Survivors of critical illness can be left with substantial physical morbidities, impacting mobility, independence of function and quality of life for several years [1–4]. Physical dysfunction begins whilst still on intensive care, with fast-developing muscle wasting and intensive care unit (ICU)-acquired weakness [5,6]. Physical rehabilitation (often referred to as early mobilisation of patients) begun during critical illness can improve short-term outcomes such as time spent in hospital and level of physical functioning [7]. However, higher intensity physical activity implemented earlier during an ICU stay does not improve outcomes and may have safety implications [8]. It is therefore uncertain when the optimal timepoint to begin rehabilitation on intensive care is. There is likely a balance between starting when it can most effectively address acutely developing physical complications, whilst not increasing safety concerns [9,10].

One key consideration for when to start rehabilitation is a patient’s cardiovascular stability, including whether they are receiving vasoactive drugs [11,12]. Indeed, cardiovascular instability has been related to adverse events recorded during rehabilitation and is regularly identified as a barrier to starting rehabilitation [13–19]. Previous expert clinical guidance has not reached agreement on the risk of rehabilitation with vasoactive drugs [11]. This is likely because there is insufficient evidence that allows clinicians to weigh benefits and risks and assess the optimal time to begin physical activity in this patient group. Current data are limited to observational studies of early physical activity, frequently focused on a cardiothoracic population, which show low adverse event rates [20–24]. There is therefore, a need for a randomised controlled trial to evaluate the relative effects of an enhanced dose of rehabilitation with patients receiving vasoactive drugs on function and safety, in comparison to standard care.

Before such a trial, a feasibility study is required to inform robust trial design, including describing what standard care currently is [25,26]. Furthermore, it is important to know the degree of clinician equipoise for randomisation of rehabilitation interventions [26] and whether a trial is acceptable to patients. Finally, further information is required over the optimal way to evaluate outcomes for this patient group.

We therefore designed a study of ICU patients receiving vasoactive drugs to describe:

1. current practice of physical rehabilitation,
2. the feasibility and acceptability of recruitment and randomisation of patients into a future trial,
3. the feasibility of measuring different outcomes.

## Methods

### Study design

**Fig 1:**
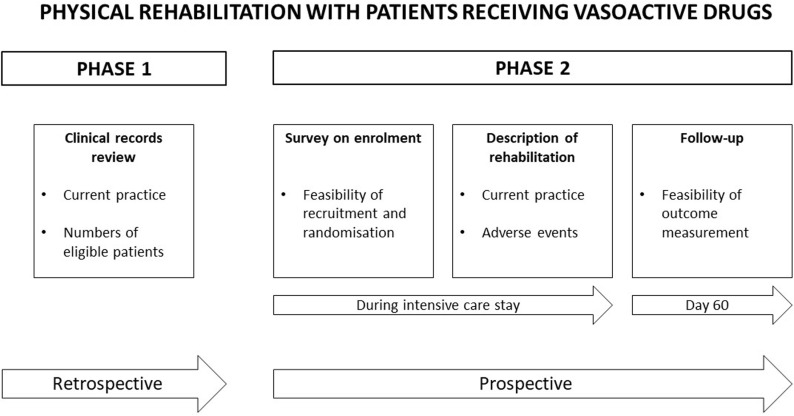
Study design overview.

A two-phase exploratory observational feasibility study was completed (Fig 1). Phase one was a retrospective, observational study utilising routinely collected data from clinical records to describe current practice and the numbers of patients eligible for inclusion in a future trial. The UK Health Research Authority approved the study and confirmed that ethical approval and participant consent was not required for this phase. Phase two was a prospective, observational study focused on measuring the feasibility of recruitment and outcome measurement. This was completed using an observational study of patients receiving vasoactive drugs, plus surveys of patients or their consultees and clinicians. The methods (including a copy of the study protocol) were registered on ClinicalTrials.gov (NCT03869541) and ethical approval was gained from Wales Research Ethics Committee 6 (18/WA/0310). In addition to the methods set out below, further data collection details are found in S1 File. For this study, ‘physical rehabilitation’ and ‘mobilisation’ were used interchangeably and included active exercise and movements in the bed, and/or active or passive mobility out of the bed, such as moving to a chair and standing up (ICU mobility scale 1–10) [27]. Success criteria indicating feasibility for future investigation were defined *a priori* and are outlined in Table 1.

**Table 1:** *A priori* feasibility success criteria.

Key feasibility outcome	Feasibility success threshold^a^
**Current practice of physical rehabilitation: Phase one**	
Number of participants taking part in physical rehabilitation whilst receiving vasoactive drugs	< 70%
**Feasibility and acceptability of recruitment and randomisation: Phase two**	
Clinician acceptability of hypothetical randomisation:	
Early v. no rehabilitation	>80%
Early rehabilitation v. standard care	>80%
Protocolised rehabilitation v. standard care	>80%
**Feasibility of outcome measurement: Phase two**	
Day 60 outcome-specific follow up rate:	
Mortality	≥ 80%
EQ-5D-5L	≥ 80%
WHODAS 2.0	≥ 80%
Physical function domain of RAND SF-36 v1	≥ 80%
6-minute walk test	≥ 80%
Feasibility of measuring baseline characteristics:	
Functional Comorbidity Index	> 98%
Clinical Frailty Scale	> 98%
WHODAS 2.0	> 98%
Adverse event tool feasibility:	
Adverse event tool loss of information	
Clinicians	≤ 10%
Researchers	≤ 10%
Time taken to complete the adverse event tool	< 5 minutes

EQ-5D-5L = EQ - 5D (5 Level) questionnaire; WHODAS 2.0 = World Health Organisation’s Disability Assessment Schedule 2.0 12-item version; RAND SF-36 v1 = RAND 36-Item Health Survey 1.0 Questionnaire.

^a^An indication to consider future investigation of physical rehabilitation with patients receiving vasoactive drugs.

### Setting

This study was carried out at a UK NHS hospital Trust in London which included three acute hospitals. Phase one used data collected by staff at two ICUs for patients admitted from June-August 2017. Data was accessed from 14^th^ September 2018 and the study ended on 30^th^ September 2019, with only the clinical care team viewing identifiable information. Phase two recruited participants from three ICUs from 6^th^ December 2018 until 10^th^ July 2019.

### Participants

#### Phase one.

Clinical records from patients admitted from the study start date were screened. ICU patients were included if they were receiving vasoactive drugs (see S1 File for definition) and if their age was greater than or equal to 18 years old.

#### Phase two.

Patient participants were included if they were admitted to the ICU and receiving vasoactive drugs, their age was greater than or equal to 18 years and they were expected to remain on the ICU for at least 24 hours after enrolment. Patients were excluded if they were expected to die imminently, if rehabilitation was contraindicated by their existing injuries, if they had neuromuscular disease or acute brain or spinal cord injury, or if they or their consultee was unable to speak English. Written informed consent was obtained for all participants. However, if patients did not have capacity, their personal or nominated consultee was approached for advice, with retrospective consent obtained when able. Recruitment prioritised patients who were not about to wean off vasoactive drugs, where follow-up was possible (e.g., patient not for discharge overseas). In addition, senior physicians, nurses and physiotherapists were recruited for a survey on acceptability of randomisation and were included if they worked on the ICU where a patient participant had been admitted. Further, ICU physiotherapists at the research sites took part in a survey exploring the feasibility of using an adverse event tool to collect data if they had used it as part of this study.

### Data collection: Current practice of physical rehabilitation

#### Phase one.

The key outcome for phase one was the number of patients mobilised (defined as 1–10 on the ICU mobility scale [27], see S1 File for details) whilst receiving vasoactive drugs. Current practice was further measured using the following secondary outcomes: patients’ trajectories were described by the number of hours receiving vasoactive drugs and the number of times vasoactive drugs were restarted after being weaned off for three hours or more. Rehabilitation whilst receiving vasoactive drugs was measured by the number of mobilisation treatment sessions; the number of vasoactive drug days (if vasoactive drugs were received at any point between 8 am and 5 pm) where rehabilitation occurred and did not occur; the highest level achieved on the ICU mobility scale [27] during each treatment session and the type and dose of vasoactive drugs received during rehabilitation.

Surrogate outcomes were also measured to describe current care and for potential use in a future trial. This included number of days from ICU admission to a) first rehabilitation treatment (included if the patient was still receiving vasoactive drugs at this point) and b) different mobility milestones. Additionally, ICU and hospital length of stay and ICU mobility scale level achieved at ICU discharge were recorded.

#### Phase two.

Phase two also collected data describing current practice by measuring rehabilitation occurring on vasoactive drugs and also surrogate outcomes as described for phase one. Additionally, days receiving vasoactive drugs was measured. The starting point for time-to-event outcomes was different between the two phases. Phase one recording started on day of admission whilst phase two started on day of enrolment, which may have been later than day one of ICU admission.

### Data collection: Feasibility and acceptability of recruitment and randomisation

#### Phase one.

Feasibility of recruitment was measured using the number of patients included within the timeframe of the study and feasibility of intervention delivery by recording when these patients would be likely to be able to start a rehabilitation intervention in a future trial. This was defined as the first time they were awake enough to participate in active rehabilitation (Richmond Agitation and Sedation Scale −2 to + 2 [28]) with no obvious contraindications between 8 am and 8 pm (see S1 File for details).

#### Phase two.

Participants or their consultee were surveyed post-enrolment about whether they would hypothetically participate in a future trial: Acceptability of three different randomisation scenarios (early rehabilitation versus no rehabilitation; early rehabilitation versus standard care rehabilitation; protocolised rehabilitation versus standard care rehabilitation) to patients or their consultee was recorded (see S1 File for survey). The early rehabilitation would start after initiation of vasoactive therapy at an earlier time point than standard care. Recruitment for a future trial was estimated by the number of participants indicating acceptability of either of the three scenarios. In addition, the consultant physician, nurse in charge and senior physiotherapist working on day of enrolment for each patient participant recruited were surveyed about acceptability of these randomisation scenarios.

### Data collection: Feasibility of outcome measurement

#### Phase two only.

Feasibility of recording candidate primary outcome measures for a future trial at day 60 post-enrolment were recorded through follow-up rates for each outcome. Mortality was recorded using site clinical records, NHS registry data or via the patient’s general practitioner. Other outcomes were recorded by phone, post or in person at the research site. These were: health-related quality of life using the EQ-5D (5 Level) questionnaire (EQ-5D-5L) [29]; disability using the World Health Organisation’s Disability Assessment Schedule 2.0 (WHODAS 2.0) 12-item version [30,31]; patient-reported physical functioning using the physical function domain of the RAND 36-Item Health Survey 1.0 Questionnaire (RAND SF-36 v1) [32] and performance-based physical functioning using the 6-Minute Walk Test (6MWT) [33,34]. Those participants who were deceased at follow-up were recorded as completing outcomes with a score of zero. Proxies were not used to complete outcomes if participants themselves were unable to complete them [35,36].

After enrolment of participants, the feasibility of measuring baseline characteristics that are associated with physical outcomes [37–40] was described by completion rates. This included the Functional Comorbidity Index [41], the Clinical Frailty Scale [42] and the WHODAS 2.0 12-item version [30]. During rehabilitation sessions, a previously developed adverse event tool [43] was used to evaluate safety of treatment sessions concurrently by the treating physiotherapist, and by the researcher using information from the clinical case notes. The loss of information, number and type of adverse events recorded were described for both users. Additionally, the adverse event rate was measured alongside the study serious adverse event rate (see S1 File for details). Finally, a survey was conducted with clinicians who used the tool to gain opinion on usability (see S2 File for survey, adapted from Hodgson et al., (2014) [27].

### Analysis

There is a lack of agreement over sample size estimates for feasibility studies with many taking a pragmatic approach [44–46]. Phase one aimed for a pragmatic sample size of 100 patients so enough information related to the number of patients mobilised on vasoactive drugs could be collected. This has previously ranged from 9% [47] to 81% [48] of patients; therefore, if the number of patients mobilised on vasoactive drugs was low in our study, our sample would still give us data to contribute to the description of standard care. Phase two aimed to recruit a maximum of 40 patients, a pragmatic sample size to maximise data for the key outcomes within the study timeframes, an approach consistent with previous exploratory studies of this type [49,50] and based upon local experience of feasible recruitment within the study time period.

Descriptive statistics were used to summarise data using IBM SPSS Statistics V.25. Continuous data were tested for normality using the Shapiro-Wilk test, then summarised as appropriate with median and interquartile range or mean and standard deviation. Categorical variables were summarised using percentages. Time to event outcomes such as length of stay were censored at day 60.

## Results

### Participants

#### Phase one.

**Fig 2:**
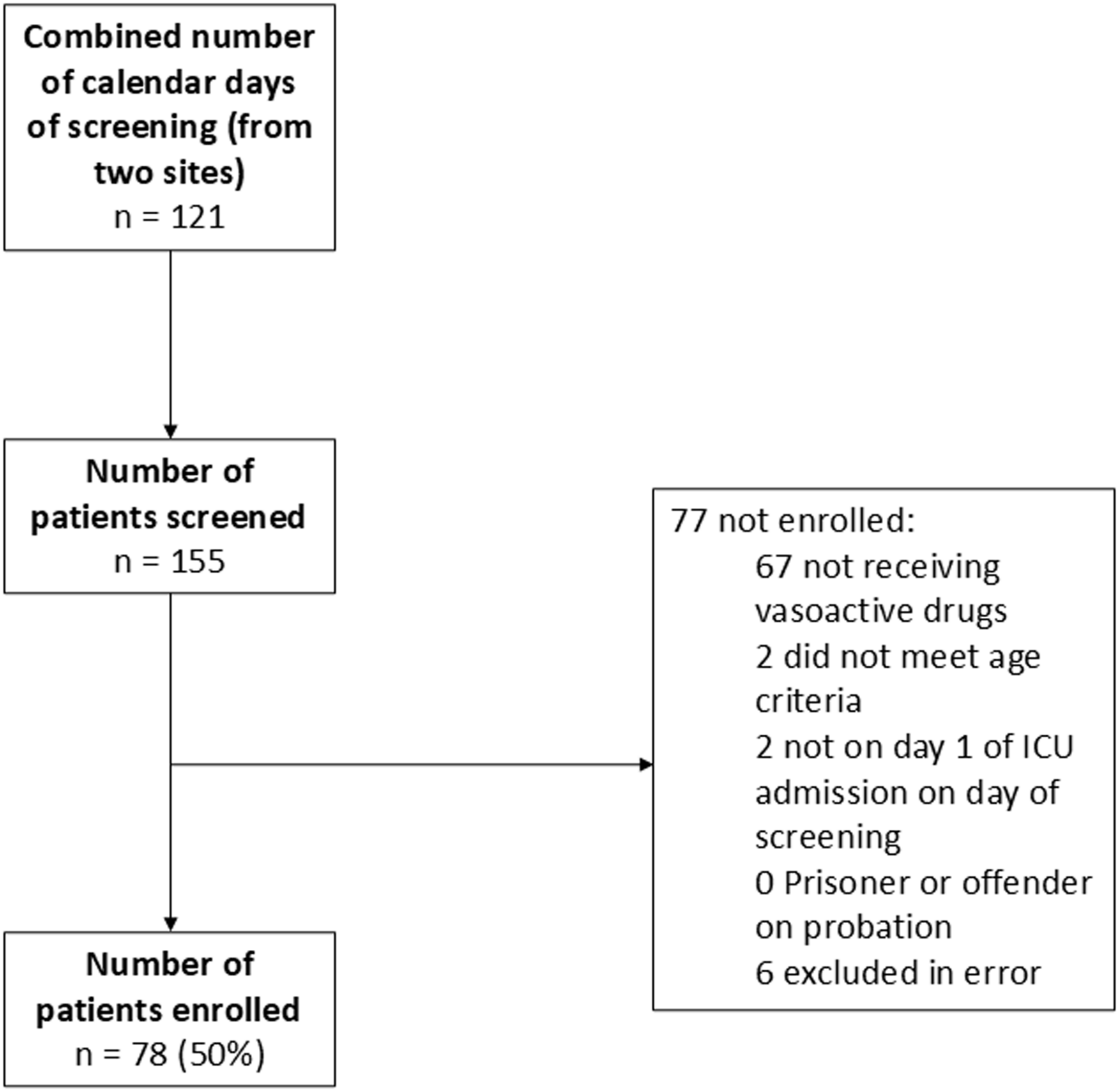
Phase one participant screening flow chart.

Seventy-eight participants were included in phase one with recruitment stopping before the sample size target of 100. Key to this decision was that sufficient data had been collected to answer study objectives. In addition, data collection was extremely time consuming and there was limited capacity for further data extraction. Details of patient screening and enrolment are in Fig 2 and participant demographics are given in Table 2.

**Table 2:** Participant demographics for the two-phase exploratory observational study: Phase 1 retrospective observational study and phase 2 prospective observational study.

	Phase 1 participants (N = 78)	Phase 2 participants (N = 40)
Age (years)	65 (53–74)	66 (54–72)
Sex: Male, n (%)	52 (67)	26 (65)
Ethnicity, n (%)		
Asian/Asian British	15 (23)^a^	0 (0)
Black/African/Caribbean/Black British	3 (5)^a^	3 (8)
White	34 (53)^a^	29 (73)
Other/not stated	12 (19)^a^	8 (20)
Weight (kg)	72 (15)^b, c^	78 (70–99)^d^
BMI	24 (4)^b, c^	26 (23–31)^e^
Reason for admission, n (%)		
Abdominal/gastrointestinal/pelvic surgery	16 (21)	12 (30)
Cardiothoracic surgery	4 (5)	0 (0)
Cardiovascular	8 (10)	6 (15)
Gastrointestinal	4 (5)	0 (0)
Neurologic	10 (13)	2 (5)
Orthopaedic surgery	0 (0)	2 (5)
Respiratory	5 (6)	3 (8)
Sepsis	10 (13)	4 (10)
Trauma	11 (14)	0 (0)
Vascular surgery	4 (5)	9 (23)
Other/mixed reasons	6 (8)	2 (5)
APACHE II	17 (13–23)	16 (12–21)
SOFA score	7 (5–10)	6 (4–9)
Requiring renal replacement therapy during admission, n (%)	15 (23)^f^	7 (18)
Number of days receiving mechanical ventilation	4 (1–13)^g^	0 (0–7)
Number of days receiving non-invasive ventilation or high flow nasal cannula oxygen	1 (0–3)^g^	0 (0–1)
Number of vasoactive drugs received on day of enrolment, n (%)		
1 vasoactive drug	69 (89)	36 (90)
2 vasoactive drugs	7 (9)	3 (8)
3 vasoactive drugs	2 (3)	1 (3)
Vasoactive drug received on day of enrolment, n (%)		
Noradrenaline	75 (96)	31 (78)
Meteraminol	5 (6)	9 (23)
Dobutamine	1 (1)	1 (3)
Adrenaline	4 (5)	0 (0)
Vasopressin	2 (3)	2 (5)
Enoximone	1 (1)	1 (3)
Milrinone	1 (1)	1 (3)

Data presented as median (interquartile range) unless otherwise indicated. kg = kilogram; BMI = body mass index; APACHE II = Acute Physiology and Chronic Health Evaluation, version II; SOFA = Sequential Organ Failure Assessment. The number of participants varied for some data and therefore the population number has been given where full data sets were not collected.

^a^n = 64; ^b^n = 55, ^c^are presented as mean (±standard deviation); ^d^n = 39; ^e^n = 35; ^f^n = 66; ^g^n = 77.

#### Phase two.

**Fig 3:**
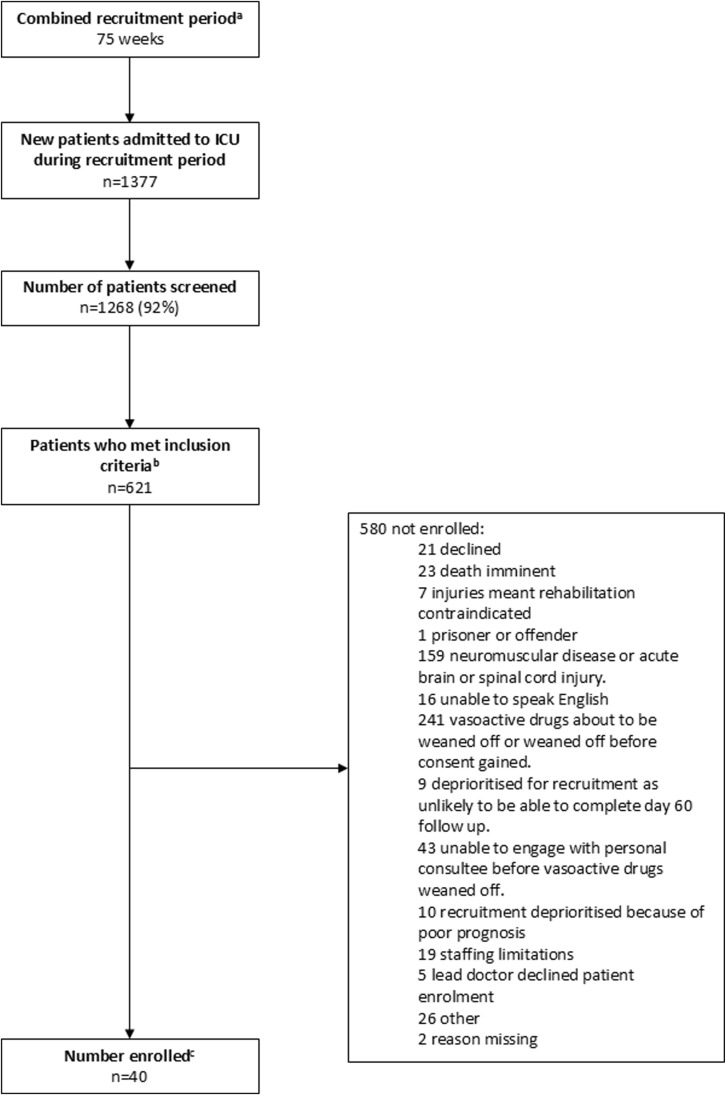
Phase two participant screening flow chart. ^a^Combined recruitment period for three research sites recruiting from December 2018 to May 2019, recruitment on any number of days within a week was counted as a week of recruitment. ^b^Due to a data collection error, there is missing data for numbers of patients who met inclusion criteria and the number not enrolled: Site 1: Data not available for first 19 days of recruitment. Site 2: Data not available for first 25 days of recruitment. Site 3: Data not available for first 15 days of recruitment. ^c^data available for whole recruitment period.

Forty patient participants receiving vasoactive drugs were enrolled (see Table 2 for demographic details). Fig 3 contains details of patient screening.

Table 3 presents a summary of main outcomes compared against the pre-defined feasibility outcomes.

**Table 3:** Results of key feasibility outcomes mapped against *a priori* feasibility success criteria.

Key feasibility outcome	Result n (%)	Feasibility success criteria
**Current practice of physical rehabilitation: Phase one**		
Number of participants taking part in physical rehabilitation whilst receiving vasoactive drugs (n = 78)	16 (21%)	< 70%
**Feasibility and acceptability of recruitment and randomisation: Phase two**		
Clinician acceptability of hypothetical randomisation		
Early v. no rehabilitation (n = 106 clinicians)	35 (33%)	>80%
Early rehabilitation v. standard care (n = 106 clinicians)	93 (88%)	>80%
Protocolised rehabilitation v. standard care (n = 107 clinicians)	88 (82%)	>80%
**Feasibility of outcome measurement: Phase two**		
Day 60 outcome-specific follow up rate:		
Mortality (n = 40)	40 (100%)	≥ 80%
EQ-5D-5L (n = 23)^a^	16 (70%)	≥ 80%
WHODAS 2.0 (n = 23)^a^	15 (65%)	≥ 80%
Physical function domain of RAND SF-36 v1 (n = 23)^a^	15 (65%)	≥ 80%
6-minute walk test (n = 23)^a^	6 (26%)	≥ 80%
Feasibility of measuring baseline characteristics (n = 40):		
Functional Comorbidity Index	32 (80%)	> 98%
Clinical Frailty Scale	38 (95%)	> 98%
WHODAS 2.0	38 (95%)	> 98%
Adverse event tool feasibility:		
Adverse event tool loss of information (n = 42 treatment sessions)		
Clinicians	23 (55%)	≤ 10%
Researchers	1 (2%)	≤ 10%
Time taken to complete the adverse event tool: number completed in < 5 minutes (n = 5 uses of the tool)	4 (80%)	< 5 minutes

EQ-5D-5L = EQ - 5D (5 Level) questionnaire; WHODAS 2.0 = World Health Organisation’s Disability Assessment Schedule 2.0 12-item version; RAND SF-36 v1 = RAND 36-Item Health Survey 1.0 Questionnaire.

^a^Follow up rate calculated as a percentage of number of participants followed up in person.

### Current practice of physical rehabilitation

#### Phase one.

Out of the 78 participants, 16 (21%) mobilised whilst receiving vasoactive drugs. From a total of 321 days receiving vasoactive drugs, physical rehabilitation occurred on 27 days (8%). The 25 physical rehabilitation sessions completed were carried out on a low infusion rate of noradrenaline (median dose of 0.05 mcg/kg/min, IQR, 0.02–0.08) with a median highest level of mobility achieved of three on the ICU mobility scale (IQR 1–5, data available for 24 treatments). Only 23% of first in-bed and 18% of first out-of-bed rehabilitation sessions occurred whilst participants were still receiving vasoactive drugs (data available for 64 and 67 participants respectively). The median duration of vasoactive drugs was 53 hours (IQR 28–100). Tables A and B in S2 File contains further data on rehabilitation received and other measures of standard care.

#### Phase two.

Data recorded describing standard care of participants, including rehabilitation received and surrogate outcomes (for example length of stay and time to mobility milestones) are contained in Tables C, D and E in S2 File. These data are broadly comparable to phase one, although a greater proportion of patients (50%) took part in rehabilitation whilst receiving vasoactive drugs and mobility milestones were achieved at an earlier time point.

### Feasibility and acceptability of recruitment and randomisation

#### Phase one.

Forty-five (58%) participants were theoretically able to participate in a physical rehabilitation intervention whilst receiving vasoactive drugs, being awake enough without contraindications to exercise at that time. Table F in S2 File contains further details of this estimate.

#### Phase two.

Twenty-five (66%) participants were enrolled before their first routine rehabilitation treatment (data available for 38 participants). Recruitment was initially facilitated through a personal consultee for 14 (35%) participants and nominated consultee for 5 (13%) participants.

When these participants or their personal consultee were surveyed at time of enrolment, 81% of participants agreed they would be willing to participant in a future trial (data available for 37 participants). When clinicians were surveyed (n = 106), 88% had clinical equipoise for the hypothetical randomisation scenario ‘early rehabilitation or standard care’ for patients and 33% had equipoise for ‘early rehabilitation or no rehabilitation’ (Table 3). When clinician and patient randomisation preference was considered together, ‘early rehabilitation or standard care’ was the most acceptable (83%, n = 143). Full details of survey responders and responses are contained in Tables G and H in S2 File.

### Feasibility of outcome measurement

#### Phase 2 only.

A 100% follow-up rate was achieved at day 60 for participant mortality outcomes. Ten weeks before the study end date it became clear it was not feasible to measure the six-minute walk test. Therefore, follow-up involving direct contact with participants was stopped as feasibility outcome conclusions were already clear, and appropriate available resources were not available for follow-up after this period; only mortality outcomes were recorded after this time. Follow-up rates for other candidate primary outcomes are therefore presented as a percentage of participants where direct contact follow-up was attempted (n = 23). None of these other outcomes were completed above the 80% pre-defined follow-up rate (Table 3). However, follow-up rates for patient-reported outcomes at this time point (70% for EQ-5D-5L; 65% for WHODAS 2.0; 65% for physical function domain of RAND SF-36 v1) were substantially higher than that for the performance-based six-minute walk test (26%). Reported reasons for non-completion of the walk test included the participant declining, reporting they would be unable to manage it and being unable to travel to the research site. Of note, for the WHODAS 2.0, six participants marked the education or employment question as not relevant. Full details of day-60 follow-up are in Table I in S2 File.

Baseline measure completion rates for the 40 participants were 95% for the Clinical Frailty Scale and WHODAS 2.0 and 80% for the Functional Comorbidity Index, which was mainly impacted by lack of availability of accurate data to calculate Body Mass Index. Further details are available in Table J in S2 File.

The adverse event tool was used to evaluate 42 rehabilitation treatments whilst receiving vasoactive drugs. It was completed less often by the treating physiotherapist following a rehabilitation session (55% loss of information) than by researchers who completed the tool at another time using data from clinical notes (2% loss of information). Although missing data impacts clinician loss of information, presumably this loss was because of the time pressures clinicians are under. A 15% adverse event rate was found, the majority consisting of hypotension, with no additional adverse events picked up by the serious adverse event measure. Ten surveyed clinicians reported excellent usability, including 100% indicating it was easy and clear to understand. Further information is contained in Tables K, L and M in S2 File.

## Discussion

This study found that rehabilitation with ICU patients happens infrequently during the time they receive vasoactive drugs. The rehabilitation that did occur was to a moderate mobility level and was often whilst the patients were receiving low doses of noradrenaline, demonstrating how clinicians perceived cardiovascular stability for mobility. Patient participants receiving vasoactive drugs indicated in principle they would be willing to participate in a future trial at a rate suggesting that recruitment is feasible. Importantly, ICU clinicians had clinical equipoise for these patients to be randomised into receiving either standard care or an early rehabilitation intervention. Outcome measurement was feasible at day-60 post-enrolment and patient-reported outcomes had a much higher completion rate than performance-based outcomes.

The detailed information gained on usual care of rehabilitation whilst receiving vasoactive drugs in phase one adds depth of insight into current practice. The finding that rehabilitation occurred on lower doses of vasoactive drugs is comparable to previous descriptions of rehabilitation with this patient group [20,21,23,25]. However, the quantity of rehabilitation whilst receiving vasoactive drugs (measured either by the number mobilised or the number of days that rehabilitation occurred) was substantially lower in phase one of this study compared with some previous work [21,23]. These differences may be explained by practice variations between the countries or other patient characteristics. For example, it is interesting to note that in our prospective study, patients underwent a greater amount of rehabilitation compared with our retrospective study. This may be explained by the lower proportion of mechanically ventilated patients recruited during the second phase. Therefore, patients receiving vasoactive drugs plus mechanical ventilation may be an important consideration for future trial plans.

The adverse event rate during rehabilitation with vasoactive drugs in phase two (15%) was higher than previous studies [21–24], who recorded adverse event rates ranging from 0.87% to 7.8%. It is difficult to know why there was a higher percentage of adverse events. Some other studies recruited patients admitted after cardiothoracic surgery [21,22] compared with our more general population. Rebel et al. [23] had a generally more comparable patient group but still found a lower number of adverse events, perhaps due to the broader definition of unsafe hypotension given in our adverse event tool. In future, direct comparison of adverse events would be facilitated by a standardised adverse event tool used across studies [43].

The high proportion of phase two participants indicating willingness to participate in a future trial is in line with acceptable recruitment proportions in previous feasibility studies of physical rehabilitation interventions [50–52]. However, comparison is limited as our recruitment estimate is based on hypothetical rather than actual recruitment. The acceptability of ‘no rehabilitation’ as a control arm to patients and clinicians was much lower than the acceptability of a ‘standard care’ comparator, which is in line with practice in recent physical rehabilitation trials [8,53].

Several candidate outcome measures have been explored. These were challenging to select as a core outcome set for physical rehabilitation of ICU patients had not been completed at the time of the study [54]. Therefore, outcomes were selected from a related outcome measure set [55] and discussion with experts to cover different characteristics of physical functioning [56]. Questionnaire-based measures (such as EQ-5D-5L and the physical function domain of the RAND SF-36) were more feasible to use than performance-based outcomes, likely because of the convenience of remote-completion [57,58]. Although difficulty in uptake of the six-minute walk test was found in our population, it should be noted that this outcome has been successfully implemented in several other studies measuring long-term outcomes of ICU survivors [1,6,59]. This difference may be due to a different follow up approach. For example, providing the option for assessment at home [1,6] or providing transport to the clinic setting [60] and by providing regular communication with participants to prompt investment in ongoing participation [60,61]. This indicates that the follow-up approach is important to ensure good follow up rates for a performance-based outcome in a future trial. Furthermore, implementation of the six-minute walk test in our study may have been impacted by the particular cohort of ICU survivors that were recruited and how they were impacted by their ICU stay. Some of our participants declined to complete the walking test as they felt they were physically unable. An additional less physically-demanding measure such as the timed up and go test [56,62] could be considered, depending on the cohort.

The low amount of rehabilitation delivered whilst patients receive vasoactive drugs and the clinicians’ equipoise supports the need for a future trial investigating the effects of rehabilitation for this subgroup of patients in an ICU. The description of current practices informs the design of a future intervention that builds upon what is delivered currently. However, further work is first required to refine the dose and timing of physical activity, which requires a structured methodological approach to design this complex intervention [9,63].

Importantly, a future trial was acceptable to patients and the preliminary indicators of recruitment can inform resource planning. Notably, we found that the recruitment was markedly faster at one hospital site. This may not have been the availability of the relevant cohort of patients, but instead it may have been the influence of key individuals who were particularly proactive research practitioners. This highlights the importance of local study champions and building of a team who value the importance of a particular study to aid recruitment, particularly in the context of multiple concurrent studies. Importantly, these champions can be from across multiple professional disciplines. Furthermore, to ensure early enrolment, this study highlighted the importance of utilising professional surrogate decision makers in the recruitment process.

Regarding outcomes, a newly developed adverse event tool was clear and understandable to clinicians and feasible to use by researchers reviewing clinical case notes. However, its completion by treating physiotherapists following a rehabilitation session led to some missing information, which could be overcome by strengthening their involvement in the study design and planning. This new tool now requires reliability and validity testing to facilitate its use in future research. Additionally, the components of the WHODAS 2.0 related to work and study were not always relevant to the patient group in this study; therefore, another measure such as the Lawton Instrumental Activities of Daily Living Scale [64] could be considered. Furthermore, future trials should include clinical outcomes measured at a timepoint closer to the intervention, such as duration of mechanical ventilation and days on ICU as these have previously been influenced by ICU rehabilitation interventions [65].

The strengths of this study lie in the in-depth description of usual care across multiple sites and the prospective assessment of future trial feasibility and acceptance. The nature of retrospective data collection relying on accuracy and availability of data from clinical records in phase one, leading to a degree of missing data, mean conclusions drawn from phase one are necessarily broad. Recruitment acceptability data is limited by the lack of detailed information about a future trial provided when participants were surveyed. Further limitations include the pragmatic sample sizes, the lack of comparison group, potential impact of confounding organ support on the safety of rehabilitation and the reduced follow-up in phase two for logistical reasons. However, the magnitude in difference in feasibility of completing questionnaires or a patient exercise test means that the main conclusions drawn are unlikely to have been different. Nevertheless, these limitations may impact the readiness to design a full trial, which should be taken into account when planning the next stage of trial development. Finally, this study did not measure whether participants had ICU-acquired weakness; therefore, it is uncertain how the presence of this condition impacted outcomes.

## Conclusions

This observational, preliminary feasibility study demonstrated low levels of rehabilitation with patients receiving vasoactive drugs. A future trial of rehabilitation is acceptable to this group of patients. Clinicians have personal equipoise for randomisation. Based on the results of this study, mortality, EQ-5D-5L and the physical functioning domain of the RAND SF-36 v1 should all be considered as outcome measures, with consideration given to the timed up and go test as a performance-based measure of physical functioning.

## Supporting information

S1 File Methods supplementary material. Including acceptability of randomisation survey and study data collection procedures.
(PDF)

S2 File Results supplementary material. Phase one and two supplementary data.
(PDF)
