# Supplementary material for: Investigating the safety of physical rehabilitation with critically ill patients receiving vasoactive drugs: An exploratory observational feasibility study
Source: PLoS One. 2025 Feb 13;20(2):e0318150. doi: 10.1371/journal.pone.0318150 (PMC11824961; doi:10.1371/journal.pone.0318150)
Supplement: S1 File — Including acceptability of randomisation survey and study data collection procedures. (PDF) [file pone.0318150.s001.pdf]

S1 File: Methods supplementary materials

Investigating the safety of physical rehabilitation with critically ill patients receiving vasoactive drugs: an exploratory observational feasibility study

Participants 2

Additional exclusion criteria for phase one and two 2

Data collection: Feasibility of recruitment and randomisation 2

Phase one: criteria for being able to start a rehabilitation intervention in a future trial 2

Phase two: survey for patient/consultee acceptability of randomisation. 3

Data collection: Feasibility of outcome measurement 5

Phase two: serious adverse event definition. 5

Study data collection procedures 5

References 7

Participants

Additional exclusion criteria for phase one and two

In addition to exclusion criteria stated in the main manuscript, patient participants were also excluded from both phases of the study if they were prisoners or offenders on probation.

Data collection: Feasibility of recruitment and randomisation

Phase one: criteria for being able to start a rehabilitation intervention in a future trial

The first time participants were awake enough to participate in active rehabilitation (Richmond Agitation and Sedation Scale[1] -2 to +2) with no obvious contraindications, for at least four successive hours, ending between 8am and 8pm.

Contraindications defined as per criteria by Woodbridge et al. (2) and Hodgson et al. (3, p.3) as ‘significant potential risk or consequences of an adverse event’. However, where there was overlap, Woodbridge et al. (2) took priority as this focused on the specific study participants who are receiving vasoactive drugs.

Phase two: survey for patient/consultee acceptability of randomisation.

Patient participant initial survey Case Report Form

Study code for patient participant: ________________

Information about respondent

1. Is the respondent the:

☐ *Patient*
☐ *Consultee*

If a consultee, please give details on relation to patient participant (e.g. friend/relative):

____

2. Time since PATIENT participant was recruited and admitted:

____

Acceptability of hypothetical RANDOMISATION into a future RCT on the mobilisation of patients receiving vasoactive drugs.

- Outline plan for future RCT as per participant information sheet.
- Ensure understanding of meaning of vasoactive drugs

	Yes	No	Unsure
1. Early physical rehabilitation v. no physical rehabilitation	☐	☐	☐
Reasons:			
2. Early physical rehabilitation v. standard care	☐	☐	☐
Reasons:			

3. 'Protocolised care' (where the decision to mobilise is based on a pre-defined risk stratification protocol) v. standard care	☐	☐	☐
Reasons:			

Other comments:

Acceptability of hypothetical RECRUITMENT into a future RCT on the mobilisation of patients receiving vasoactive drugs.

	Yes	No	Unsure
4. Did patient/consultee know patient was receiving vasoactive drugs before being approached for the exploratory observational study?	☐	☐	☐
Comments:			
5. Acceptability of hypothetical RECRUITMENT into a future RCT on the mobilisation of patients receiving vasoactive drugs (with one of the randomisation scenarios above)	☐	☐	☐
Reasons:			

Data collection: Feasibility of outcome measurement

Phase two: serious adverse event definition.

Serious adverse events were defined as per standard UK Research Ethics Service and Health Research Authority definitions [4, 5].

Study data collection procedures

Data definitions:

- Vasoactive drugs were defined as continuous intravenous infusions of vasopressors or positive inotropes.
- “In-bed” rehabilitation: Intensive care unit (ICU) mobility scale level 1 [3, p. 2, 6].
- “Out-of-bed” rehabilitation: ICU mobility scale levels 2-10 [3, p. 2, 6].
- Day receiving vasoactive drugs: If vasoactive drugs received at any point between 8am and 5pm.
- Days receiving vasoactive drugs where rehabilitation occurred: Defined as above, where rehabilitation occurred at any point between 8am and 5pm.
- Number of times patient restarted on vasoactive drugs: Only if three or more hours between drugs stopping and restarting.

Accuracy was maintained through clarifying any uncertainty over data points with other sources in the clinical record. Any patient readmission to ICU within 48 hours were included as the same ICU admission. Fluctuations in vasoactive drug dose were mitigated by averaging four hours of drug dose at a particular data timepoint. Rehabilitation treatments were only recorded from therapist documentation and treatments were quantified by the highest mobility level achieved. Additionally, for phase two, the vasoactive drug dose on day of enrolment was taken at midday.

Phase one data was collected retrospectively and therefore relied on the accuracy of the clinical records and data being available that addressed the pre-defined outcomes, leading to a degree of missing data. This particularly impacted data for vasoactive drugs received and/or the time point of being ready to begin rehabilitation. The following steps were taken to minimise study bias.

For phase one, data collection was carried out by two site clinicians who received initial training in the study protocol and by completing initial data collection with HRW, allowing the data extraction tool to be informally piloted and any study definitions to be clarified. Formal data monitoring took place for six participants. Early in the formal data monitoring process, eight data point errors were identified for one participant, which were corrected and previously collected data was reviewed to ensure these errors were not made systematically. For the other five participants' data included in the monitoring process during data collection, an average of two data point errors per participant were found.

References

1. Sessler CN, Gosnell MS, Grap MJ, Brophy GM, O'Neal PV, Keane KA, et al. The Richmond Agitation-Sedation Scale: validity and reliability in adult intensive care unit patients. Am J Respir Crit Care Med. 2002;166(10):1338-44. doi: 10.1164/rccm.2107138. PubMed PMID: 12421743.
2. Woodbridge HR, McCarthy CJ, Jones M, Willis M, Antcliffe DB, Alexander CM, et al. Assessing the safety of physical rehabilitation in critically ill patients: a Delphi study. Crit Care. 2024;28(1):144. doi: 10.1186/s13054-024-04919-x. PubMed PMID: 38689372.
3. Hodgson CL, Stiller K, Needham DM, Tipping CJ, Harrold M, Baldwin CE, et al. Expert consensus and recommendations on safety criteria for active mobilization of mechanically ventilated critically ill adults. Crit Care. 2014;18(6):658. doi: 10.1186/s13054-014-0658-y. PubMed PMID: 25475522.
4. European Medicines Agency. Guideline for good clinical practice E6(R2) [Internet]. 2016 [cited 2nd April 2024]. Available from: https://www.ema.europa.eu/en/documents/scientific-guideline/ich-e-6-r2-guideline-good-clinical-practice-step-5_en.pdf.
5. UK Health Departments Research Ethics Service. Standard Operating Procedures for Research Ethics Committees Version 7.4 [Internet]. 2019 [cited 2nd March 2021]. Available from: https://www.hra.nhs.uk/about-us/committees-and-services/res-and-recs/research-ethics-committee-standard-operating-procedures/.
6. Hodgson C, Needham D, Haines K, Bailey M, Ward A, Harrold M, et al. Feasibility and inter-rater reliability of the ICU Mobility Scale. Heart Lung. 2014;43(1):19-24. doi: 10.1016/j.hrtlng.2013.11.003. PubMed PMID: 24373338.
