# Supplementary material for: Investigating the safety of physical rehabilitation with critically ill patients receiving vasoactive drugs: An exploratory observational feasibility study
Source: PLoS One. 2025 Feb 13;20(2):e0318150. doi: 10.1371/journal.pone.0318150 (PMC11824961; doi:10.1371/journal.pone.0318150)
Supplement: S2 File — Phase one and two supplementary data. (PDF) [file pone.0318150.s002.pdf]

S2 File: Results supplementary materials

Investigating the safety of physical rehabilitation with critically ill patients receiving vasoactive drugs: an exploratory observational feasibility study

Current practice of physical rehabilitation 3

Phase 1 3

Table A: Participant vasoactive drug and rehabilitation data 3

Table B: Phase 1 surrogate outcomes 4

Phase 2 4

Table C: Duration of vasoactive drugs and number of rehabilitation treatments 4

Table D: Characteristics of physical rehabilitation treatments whilst receiving vasoactive drugs 5

Table E: Phase two surrogate outcomes 5

Feasibility of recruitment and randomisation 6

Phase 1 6

Table F: Characteristics of participants on theoretical day they were first able to participate in physical rehabilitation whilst receiving vasoactive drugs. 6

Phase 2 7

Table G: Randomisation survey responder details 7

Table H: Randomisation survey results 8

Feasibility of outcome measurement 9

Phase 2 9

Table I: Day 60 outcome measures 9

Table J: Patient participant baseline variables 10

Table K: Adverse event tool feasibility 11

Table L: Adverse event tool usability survey participant demographics 11

Table M: Adverse event tool usability survey quantitative results 12

References 12

Current practice of physical rehabilitation

Phase 1

Table A: Participant vasoactive drug and rehabilitation data

	Participants (N=78)
Duration of vasoactive drugs (hours), median (IQR)	53 (28-100)
Number of times vasoactive drugs were restarted, median (IQR)	0 (0-2)
Number of in-bed only rehabilitation treatment sessions whilst receiving vasoactive drugs received, n (%)	
0	73 (94)
1	4 (5)
5	1 (1)
Number of out-of-bed rehabilitation treatment sessions whilst receiving vasoactive drugs received, n (%)	
0	65 (83)
1	11 (14)
2	1 (1)
3	1(1)
Total number of rehabilitation treatment sessions receiving vasoactive drugs, n (%)	
0	62 (80)
1	12 (15)
2	2 (3)
3	1 (1)
6	1 (1)
Number of vasoactive drug days where in-bed only rehabilitation occurred, n (%)	
0	73 (94)
1	4 (5)
6	1 (1)
Number of vasoactive drugs days where out-of-bed rehabilitation occurred, n (%)	
0	65 (83)
1	10 (13)
2	2 (3)
3	1 (1)
Total number of vasoactive drug days where rehabilitation occurred, n (%)	
0	62 (80)
1	12 (15)
2	2 (3)
3	1 (1)
8	1 (1)
Total number of vasoactive drug days where no rehabilitation occurred, median (IQR)	3 (1-5)

IQR= Interquartile range

Table B: Phase 1 surrogate outcomes

	Participants (N=78)
Time to first in-bed rehabilitation treatment (days) (n=64), median (IQR)	6 (3-16)
First in-bed rehabilitation treatments that occurred whilst still receiving vasoactive drugs (n=64), n (%)	15 (23)
Time to first out-of-bed rehabilitation treatment (days) (n=67), median (IQR)	7 (3-18)
First out-of-bed rehabilitation treatments that occurred whilst still receiving vasoactive drugs (n=67), n (%)	12 (18)
Time to first sitting out of bed (days) (n=65), median (IQR)	7 (3-19)
Time to first stand (days) (n=58), median (IQR)	9 (4-22)
Time to first walk (days) (n=54), median (IQR)	10 (6-25)
ICU mobility scale at ICU discharge (n=73), median (IQR)	3 (0-6)
ICU length of stay (days) (n=73), median (IQR)	7 (4-19)
Hospital length of stay (days) (n=71), median (IQR)	24 (15-41)

Starting point of time-to-event outcomes is the day of ICU admission.
IQR= Interquartile range
If participant did not experience event (e.g. first walk) then they are not included in the analysis.

Phase 2

Table C: Duration of vasoactive drugs and number of rehabilitation treatments

	Participants (N=40)
Number of days receiving vasoactive drugs, median (IQR)	3 (2-5)
Number of in-bed only rehabilitation treatment sessions whilst receiving vasoactive drugs, n (%)	
0	32 (80)
1	6 (15)
2	2 (5)
Number of out-of-bed rehabilitation treatment sessions whilst receiving vasoactive drugs, n (%)	
0	24 (60)
1	11 (28)
2	3 (8)
6	1 (3)
9	1 (3)
Total number of rehabilitation treatment sessions receiving vasoactive drugs, n (%)	
0	20 (50)
1	12 (30)
2	5 (13)
3	1 (3)
8	1 (3)
9	1 (3)

IQR= Interquartile range

Table D: Characteristics of physical rehabilitation treatments whilst receiving vasoactive drugs

	**Physical rehabilitation sessions carried out whilst receiving vasoactive drugs (N=42)**
Highest level of ICU mobility scale achieved, median (IQR)	4 (2-6)
Number of vasoactive drugs received during rehabilitation	
1, n (%)	40 (95)
2, n (%)	2 (5)
Number receiving	
Noradrenaline, n (%)	30 (71)
Dose (mcg/kg/min), median (IQR)	0.05 (0.02-0.10)
Metaraminol, n (%)	12 (29)
Dose (mg/hr), mean (±SD)	2.02 (0.97)
Enoximone, n (%)	2 (5)
Dose (mcg/kg/min), mean (±SD)	4.17 (1.97)

IQR= Interquartile range; mcg/kg/min= micrograms/kilogram/minute; mg/hr=milligrams/hour; SD=standard deviation

Table E: Phase two surrogate outcomes

	**Participants (N=40)**
Time to first in-bed rehabilitation treatment (days) (n=29), median (IQR)	2 (1-7)
Number where event occurred before enrolment therefore excluded from above analysis, n (%)	7 (18)
First in-bed rehabilitation treatments that occurred whilst still receiving vasoactive drugs (n=29), n (%)	16 (55)
Time to first out-of-bed rehabilitation treatment (days) (n=31), median (IQR)	2 (1-7)
Number where event occurred before enrolment therefore excluded from above analysis, n (%)	5 (13)
First out-of-bed rehabilitation treatments that occurred whilst still receiving vasoactive drugs (n=31), n (%)	14 (45)
Time to first sit out of bed (days) (n=33), median (IQR)	5 (2-10)
Number where event occurred before enrolment therefore excluded from above analysis, n (%)	2 (5)
Time to first stand (days) (n=31), median (IQR)	3 (2-11)
Number where event occurred before enrolment therefore excluded from above analysis, n (%)	3 (8)
Time to first walk (days) (n=28), median (IQR)	8 (4-21)
Number where event occurred before enrolment therefore excluded from above analysis, n (%)	0 (0)
ICU mobility scale at ICU discharge (n=38), median (IQR)	5 (3-6)
ICU length of stay (days), median (IQR)	7 (4-12)
Hospital length of stay (days), median (IQR)	16 (9-32)

Starting point of time-to-event outcomes is the day of enrolment (median day 2 of ICU stay (IQR 2-3).
Follow-up censored at day 60: This happened for one participant for time to first walk and two participants for hospital length of stay.
If event occurred before participant enrolment or after participant discharged, these participants were excluded from this analysis. IQR= Interquartile range.

Feasibility of recruitment and randomisation

Phase 1

Table F: Characteristics of participants on theoretical day they were first able to participate in physical rehabilitation whilst receiving vasoactive drugs.

	**Patients eligible for participation in rehabilitation, N = 45***
Day of ICU stay, median (IQR)	2 (1-3)
RASS score, median (IQR)	0 (-1 to 0)
Number receiving mechanical ventilation, n (%)	19 (42)
Number receiving non-invasive ventilation or high flow nasal cannula oxygen, n (%)	7 (16)
Number receiving renal replacement therapy, n (%)	9 (20)
Number of vasoactive drugs received, n (%)	
1	44 (98)
2	1 (2)
Number receiving	
Noradrenaline, n (%)	45 (100)
Dose (mcg/kg/min), median (IQR)	0.07 (0.04-0.13)
Dobutamine, n (%)	1 (2)
Dose (mcg/kg/min)	3.94

IQR= Interquartile range; RASS= Richmond Agitation-Sedation Scale; mcg/kg/min= micrograms/kilogram/minute
*65 patients recorded as awake with no contraindications, 20 of these not receiving vasoactive drugs at this timepoint (data missing for seven participants). Six participants did not reach this timepoint at all because 5 were for palliative/end of life treatment, 1 was transferred to another hospital not included in the study.

Phase 2

Table G: Randomisation survey responder details

For each of the 40 patient participants, survey completed by: n (%)	
Patient themselves*	23 (58)
Patient personal consultee*	14 (35)
Doctor	32 (80)
Nurse	35 (88)
Physiotherapist	40 (100)
**Clinician participant demographics**	**Participants (N=50)**
Profession, n (%)	
Lead Doctor	17 (34)
Nurse	26 (52)
Physiotherapist	7 (14)
Nurse and physiotherapist seniority, (%) (n=32)	
Team leader	13 (41)
Senior clinician	19 (59)
ICU experience (years) (n=48), median (IQR)	11 (5-20)
Healthcare experience (years) (n=48), median (IQR)	16 (9-26)

*for 3 of the 40 patient participants, a randomisation survey was not completed by either the patient or their consultee. IQR= Interquartile range

Table H: Randomisation survey results

For the 40 patient participants, survey completed by: n (%)	Number who agreed in principle to hypothetical randomisation scenario: Early v. no rehabilitation	Early rehabilitation v. standard care	Protocolised rehabilitation v. standard care
**Patient/personal consultee (n=37)**			
**Yes**	15 (41)	25 (68)	24 (67)*
**No**	13 (35)	7 (19)	5 (14)*
**Unsure**	9 (24)	5 (14)	7 (19)*
**Doctor (n=32)**			
**Yes**	11 (34)	30 (94)	28 (88)
**No**	19 (59)	1 (3)	1 (3)
**Unsure**	2 (6)	1 (3)	3 (9)
**Nurse (n=35)**			
**Yes**	18 (53)^	28 (82)^	27 (77)
**No**	15 (44)^	3 (9)^	1 (3)
**Unsure**	1 (3)^	3 (9)^	7 (20)
**Physiotherapist (n=40)**			
**Yes**	6 (15)	35 (88)	33 (83)
**No**	32 (80)	4 (10)	7 (18)
**Unsure**	2 (5)	1 (3)	0 (0)
**All clinicians**	n=106	n=106	n=107
**Yes**	35 (33)	93 (88)	88 (82)
**No**	66 (62)	8 (8)	9 (8)
**Unsure**	5 (5)	5 (5)	10 (9)
**All clinicians + patients/consultees**	n=143	n=143	n=143
**Yes**	50 (35)	118 (83)	112 (78)
**No**	79 (55)	15 (10)	14 (10)
**Unsure**	14 (10)	10 (7)	17 (12)

All data n (%)

*data available for 36 patients or consultees.

^data available for 34 nurses

Feasibility of outcome measurement

Phase 2

Table I: Day 60 outcome measures

	Participants (N=40)
**Mortality**	
Outcome completed? n (%)	40 (100)
Day post-enrolment that follow up occurred, median (IQR)	66 (63-71)
Number of patients alive at day 60, n (%)	35 (88)
**EQ-5D-5L**	
Outcome completed? n (%)	16 (40)^
Day post-enrolment that follow up occurred, median (IQR)	69 (64-72)
Reasons not completed, n (%)	
Non-response	2 (5)
Not feasible – participant at another healthcare facility	2 (5)
Follow-up after end date of in-person follow up	17 (43)
Participant declined	3 (8)
EQ-5D-5L index value / utility score, median (IQR)#	0.596 (0.000-0.780)
EQ-5D-5L VAS, median (IQR)#	55 (0-79)
Follow-up rate of participants included in in-person follow up (n=23), n (%)	16 (70)
**WHODAS 2.0**	
Outcome completed? n (%)	15 (38)^
Day post-enrolment that follow up occurred, median (IQR)	69 (64-72)
Reasons not completed, n (%)	
Non-response	2 (5)
Not feasible – participant at another healthcare facility	2 (5)
Follow-up after end date of in-person follow up	17 (43)
Participant declined	3 (8)
Lack of questionnaire-specific capacity	1 (3)
WHODAS 2.0 score#, median (IQR)	16.67% (0-36.36%)
Follow-up rate of participants included in in-person follow up (n=23), n (%)	15 (65)
**Physical function domain of RAND SF-36 v1**	
Outcome completed? n (%)	15 (38)^
Day post-enrolment that follow up occurred, median (IQR)	69 (64-72)
Reasons not completed, n (%)	
Non-response	2 (5)
Not feasible – participant at another healthcare facility	2 (5)
Follow-up after end date of in-person follow up	17 (43)
Participant declined	3 (8)
Lack of questionnaire-specific capacity	1 (3)
SF-36 v1 physical function domain score, median (IQR)#	20 (0-50)
Follow-up rate of participants included in in-person follow up (n=23), n (%)	15 (65)
**6-minute walk test+**	
Outcome completed? n (%)	6 (15)^
Day post-enrolment that follow up occurred, median (IQR)	66 (63-71)
Reasons not completed, n (%)	
Non-response	1 (3)
Not feasible – participant at another healthcare facility.	2 (5)
Follow-up after end date of in-person follow up	17 (43)

Participant declined	5 (13)
Patient reports they will be unable to manage walking test	4 (10)
Participant cannot travel to research site	4 (10)
Other	1 (3)
Follow-up rate of participants included in in-person follow up (n=23), n (%)	6 (26)

^Includes 5 participants deceased at time of follow-up.
#Includes scores of zero for 5 participants who were deceased at time of follow-up.
+6-minute walk distance not reported as test was only completed by one survivor.
IQR= Interquartile range; EQ-5D-5L= EQ - 5D (5 Level) questionnaire; VAS=visual analogue scale; WHODAS 2.0= World Health Organisation's Disability Assessment Schedule 2.0 12-item version; RAND SF-36 v1= RAND 36-Item Health Survey 1.0 Questionnaire

Table J: Patient participant baseline variables

	Participants (N=40)
**Clinical Frailty Scale**	
Outcome completed? n (%)	38 (95)
Clinical frailty scale score (n=38), n (%)	
1 – Very fit	8 (21)
2 – Well, active occasionally	6 (16)
3 – Well, not regularly active	12 (32)
4 – Vulnerable	3 (8)
5 – Mildly frail	5 (13)
6 – Moderately frail	2 (5)
7 – Severely frail	2 (5)
**Functional Comorbidity Index**	
Outcome completed? n (%)	32 (80)
Number incomplete because BMI unavailable	5
Number incomplete because of insufficient data	1
Number not completed at all	2
Functional Comorbidity Index count (n=38*), median (IQR)	1 (1-2)
**World Health Organization Disability Assessment Schedule 2.0 (WHODAS 2.0) – 12-item version**	
Outcome completed? n (%)	38 (95)
WHODAS 2.0 score (n=38), median (IQR)+	22.92% (9.90-35.94%)
Version used (n=38)	
Interview	23 (61)
Proxy	15 (40)

*includes participants with complete and incomplete functional comorbidity scores.
+Includes score for one participant with one missing value which was scored using the mean of the other items for that participant. In addition, items marked not applicable twice for 'walking a long distance', once for 'joining in community activities' and once for 'dealing with people you do not know'.
IQR= Interquartile range; BMI=body mass index;

Table K: Adverse event tool feasibility

	Rehabilitation treatment sessions (N=42)	
	**Clinician use of tool**	**Researcher use of tool**
Tool completed, n (%)	19 (45)	41 (98)
Adverse events, n (%)	3 (15)*	6 (15)#
Type of adverse event, n (%)		
Hypotension	3 (100)	5 (83)
Desaturation causing symptoms	0 (0)	1 (17)
Adverse event classification		
Stopped rehabilitation, n	2^	5
Did not stop rehabilitation, but lead to further consequences, n	0	1
Serious consequences (increased length of stay, surgery, death), n	0	0
Serious adverse events, n (%)	[Not measured by clinicians]	0 (0)

* [n=20] calculated from 19 complete adverse event tools, plus one which was marked as incomplete as although it clearly recorded a specific adverse event, the classification of the adverse event not completed.
# [n=41]
^ Data available for 2 out of 3 adverse events

Table L: Adverse event tool usability survey participant demographics

	**Participants (N=10)**
Physiotherapy profession, n (%)	10 (100)
Seniority (n=9), n (%)	
Clinical lead and team leaders	5 (56)
Senior clinician	2 (22)
Junior clinician	2 (22)
ICU experience (years) (n=9), mean (±SD)	6 (5)
Healthcare experience (years) (n=9), mean (±SD)	9 (6)

SD=standard deviation

Table M: Adverse event tool usability survey quantitative results

	Participants (N=10)		
	Yes	No	Unsure
Is the adverse event tool clear, easy to understand and unambiguous? n (%)	10 (100)	0 (0)	0 (0)
Does the adverse event tool indicate what the tool is about and its overall purpose? n (%)	9 (90)	1 (10)	0 (0)
Will people filling out the tool know how to do so? n (%)	8 (80)	0 (0)	2 (20)
Are the different adverse events specified in the tool adequately defined? n (%)	10 (100)	0 (0)	0 (0)
Are any of the adverse events specified in the tool irrelevant, misleading or superfluous? n (%)	0 (0)	9 (90)	1 (10)
Are any of the adverse events specified offensive or otherwise inappropriate? n (%)	0 (0)	10 (100)	0 (0)
Are any adverse events specified unnecessary or repetitive? n (%)	0 (0)	10 (100)	0 (0)
Is the adverse event tool an appropriate length? n (%)	10 (100)	0 (0)	0 (0)

Survey design adapted from Hodgson et al. (1)

References

1. Hodgson C, Needham D, Haines K, Bailey M, Ward A, Harrold M, et al. Feasibility and inter-rater reliability of the ICU Mobility Scale. Heart Lung. 2014;43(1):19-24. doi: 10.1016/j.hrtlng.2013.11.003. PubMed PMID: 24373338.
